# The Profession in Glasgow

**Published:** 1861-11

**Authors:** 


THE PROFESSION IN GLASGOW.—"In no important town of the empire is the profession so poorly represented, as far as numbers are concerned, as in Glasgow. While the average of other cities, as Edinburgh, Manchester, Dublin, Belfast, etc., gives one medical attendant to every thousand persons, Glasgow can only show one for every two thousand. But this fact will cease to be wondered at when it is considered that in scarcely any—the smallest or poorest—village of Scotland are medical men so wretchedly ill paid as in rich, prosperous, flourishing Glasgow." —*Letter in Lancet.*

